# Hybrid Biocomposites Based on Chitosan/Gelatin with Coffee Silverskin Extracts as Promising Biomaterials for Advanced Applications

**DOI:** 10.3390/polym17233194

**Published:** 2025-11-30

**Authors:** Argyri-Ioanna Petaloti, Dimitris S. Achilias

**Affiliations:** Laboratory of Polymer and Color Chemistry and Technology, Department of Chemistry, Aristotle University of Thessaloniki, 54124 Thessaloniki, Greece

**Keywords:** chitosan, gelatin, coffee silverskin, antioxidant activity, biopolymer films, sustainable materials

## Abstract

Biopolymers such as chitosan and gelatin are emerging as leading alternatives to traditional plastic packaging due to their enhanced capabilities and biodegradability. Blends of chitosan and gelatin combine chitosan’s antimicrobial and film-forming properties with gelatin’s biocompatibility and flexibility. These biomaterials possess tunable mechanical, biological, and physicochemical properties, making them suitable for biomedical, pharmaceutical, food packaging, environmental, and agricultural applications. This study investigates the preparation and characterization of composite biopolymer films based on chitosan and gelatin, incorporating coffee silverskin extract (SSE) as a natural bioactive additive. Coffee silverskin, a by-product of coffee roasting, is rich in phenolic compounds and demonstrates notable antioxidant potential. The objective of this work was to enhance the antioxidant, mechanical, and physicochemical properties of chitosan–gelatin films through the integration of SSE. The biocomposite materials were prepared using solvent casting, followed by extensive characterization techniques, including Fourier-transform infrared spectroscopy, scanning electron microscopy, differential scanning calorimetry, and UV–Vis spectroscopy. Additionally, color measurements, mechanical properties, and physicochemical properties were assessed. The transmission rates of oxygen and water vapor were also examined, along with the antioxidant activity of the films. The inclusion of coffee silverskin extract facilitated intermolecular interactions between the polymer chains, resulting in improved structural integrity. Furthermore, films containing CSE exhibited enhanced antioxidant activity (up to 28.43% DPPH radical scavenging activity), as well as improved water vapor barrier properties and mechanical strength compared to the pure chitosan–gelatin. The films showed a yellowish appearance. There was a noticeable reduction in the rate of oxygen transmission through the films as well. These results highlight the potential of coffee silverskin as a sustainable source of functional compounds for the development of bioactive materials suited for biodegradable packaging and biomedical applications.

## 1. Introduction

Biopolymers have garnered significant attention as a viable substitute for biomedical, agricultural, and packaging materials derived from petroleum products [1]. Two of the most common natural polymers used in food and pharmaceutical and other product applications [2] are gelatin (protein) and chitosan (polysaccharide), which have emerged as viable alternatives to non-degradable petroleum-based plastics [3]. These two polymers are derived from natural and renewable resources, making them non-toxic, biodegradable, and biocompatible, commonly employed as coatings and films in the food industry [4]. Specifically, chitin, the second most common polysaccharide found in nature, after cellulose, is the starting material for obtaining chitosan (Chi). Chitosan, is a linear polymer made up of β-(1–4)-2-acetamido-d-glucose and β-(1–4)-2-amino-d-glucose units that is produced from chitin by partial deacetylation in an alkaline environment [5,6]. Chitosan has been identified as a superior film forming material [7]. Films made from chitosan exhibit a specific permeability to gases, along with favorable mechanical characteristics. Nevertheless, the high permeability of chitosan films to water vapor poses a significant limitation to their utility, as effective regulation of moisture transfer is a crucial attribute for many food products, particularly in humid conditions [5]. Furthermore, the mechanical and antimicrobial properties of pure chitosan film do not meet the standards for food packaging [8,9]. Gelatin (Gel) is another widely accessible biopolymer, derived from diverse sources [10]. Due to its favorable qualities, including low toxicity, cost-effectiveness, film-forming capacity, and biodegradability, Gel—a water-soluble protein produced by hydrolyzing animal collagen—has been recognized as a viable alternative for the creation of food packaging films [11]. However, pure gel films’ low mechanical strength, high solubility, lack of barrier qualities, and weak antibacterial and antioxidant capabilities severely limit their application in the food packaging industry [12].

Combining gelatin with other biopolymers, such as chitosan, is a simple and efficient way to overcome the inherent limits in its mechanical and barrier properties [13]. These films of chitosan (Chi) and gelatin (Gel) exhibit superior mechanical, thermal, barrier, and physicochemical characteristics in comparison to films composed of a single biopolymer, such as either Chi or Gel [14]. Functional groups like -NH_2_ and -OH may be responsible for chitosan’s ability to integrate other molecules into its structure [15]. Reinforcement of the antioxidant and antimicrobial properties of these films has been created by including strengthening ingredients, such as ZnO, Ag, and NiO nanoparticles [14,16], ethanolic extracts from plants (boldo of Chile, guarana, cinnamon, and rosemary) [17], coffee leaf extract [12], and Dracocephalum kotschyi Boiss essential oil [18]. Chitosan/gelatin blends are widely studied because they combine chitosan’s antimicrobial and film-forming abilities with gelatin’s biocompatibility and flexibility, resulting in materials with tunable mechanical, biological, and physicochemical properties. They are used in biomedical, pharmaceutical, food packaging, environmental, and agricultural applications. Specifically, their biomedical applications include the following. Wound dressings: Chitosan provides antibacterial activity, while gelatin supports cell adhesion and tissue regeneration. The blends maintain a moist healing environment and can be loaded with antimicrobial agents, growth factors, or plant extracts [19,20,21,22,23]. Drug delivery systems: They are used for controlled release of drugs, proteins, or antioxidants due to their biocompatibility and degradability [20,24,25,26]. Tissue engineering scaffolds: They can serve as 3D matrices for cell growth and differentiation, especially in cartilage, bone, and skin regeneration [27,28]. Antimicrobial coatings: They can be applied on medical devices or implants to prevent biofilm formation and infection [29,30,31]. Furthermore, the food industry is actively seeking packaging materials that possess antibacterial properties, are biocompatible, and can prolong the shelf life of food [32].

In the framework of the circular economy and the “zero waste” principle, many papers have been published recently on the evaluation of the by-products and/or wastes created during the manufacturing and processing of many food items. Given that over half of the coffee fruit is not utilized in the production of commercially available green coffee and is consequently discarded during the processing phase, it becomes intriguing to explore potential applications for these residual by-products [33]. Coffee silverskin (CS) is a thin tegument that directly covers the coffee seed. During the process of roasting, coffee beans undergo expansion, resulting in the detachment of a delicate layer, which subsequently becomes the primary by-product of the coffee roasting industries [34]. It serves as a valuable source of numerous bioactive compounds that can be isolated and subsequently utilized in food, cosmetics, and pharmaceuticals. CS contains several significant bioactive compounds, including chlorogenic acids (1–6%), caffeine (0.8–1.25%), and melanoidins (17–23%), which are formed because of the Maillard reaction during the roasting process [35].

In the current investigation, solution casting was used to create chitosan/gelatin polymer films with of coffee silverskin extract (1% and 2%). Their mechanical, thermal, morphological, physico-chemical, and antioxidant characteristics were then examined. The purpose of this investigation is to demonstrate how adding CS extract can enhance the biocomposite films’ mechanical, barrier, and antioxidant activity, making them appropriate for advanced applications, such as food packaging.

## 2. Materials and Methods

### 2.1. Materials

Coffee silverskin (CS), derived from the roasting of 100% Arabica coffee varieties, was supplied by AVEK S.A. (Thessaloniki, Greece). Chitosan (100–300 cps) of low molecular weight, code: GP7325, degree of deacetylation > 90%, derived from shrimp shell was obtained from Glentham Life sciences (Corsham, UK). Gelatin from bovine skin, Type B G6650 was purchased from sigma aldrich (Rockville, MD, USA). Glacial acetic acid (purity > 99%) CL00.0116.250 was from CHEM-LAB (Zedelgem, Belgia).

### 2.2. Preparation of Coffee-Silverskin Extracts

Using a mixture grinder, the coffee silverskin was first ground, and then it was twice washed with distilled water. Following that, it was dried in a vacuum oven for 24 h at 60 °C. To obtain an aqueous extract of the coffee silverskin powder, the hot water extraction method was finally used. This procedure involved adding 1 g of coffee silverskin powder (1% *w*/*v* extract) to 100 mL of distilled water in a volumetric flask. After 20 min of heating to 60 °C, the resultant mixture was filtered using filter paper. To create a 2% *w*/*v* extract, the identical process was carried out again.

### 2.3. Preparation of Hybrid Films

**Figure 1:**
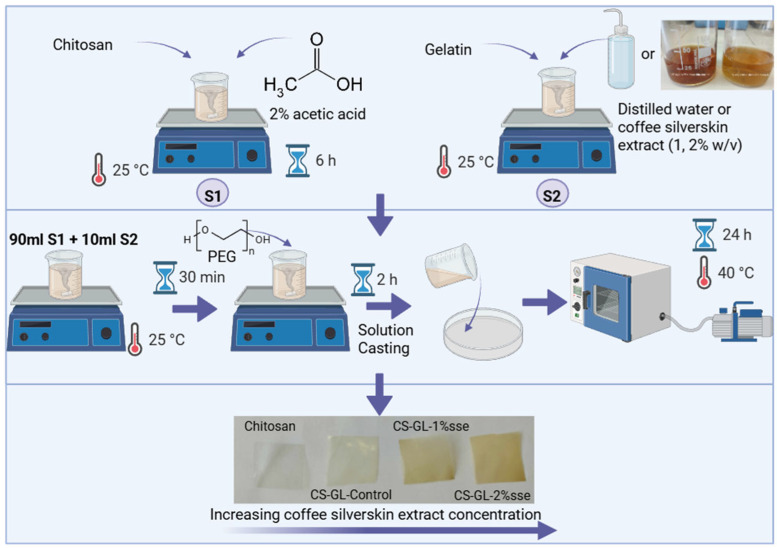
Development scheme of biocomposite chitosan–gelatin hybrid membranes with aqueous extracts of coffee silverskin. Created in BioRender. Petaloti, A. (2025). https://BioRender.com/w76qcxj.

The experimental method was adopted by Rahman et al. [36] and Kumar S. et al. [16] with changes (Figure 1). At first, two methods were devised, namely Solution-1 (S1) and Solution-2 (S2). S1 entailed the dissolution of 2 g of chitosan in 100 mL of 2% (*v*/*v*) acetic acid with the use of a magnetic stirrer for 6 h at ambient temperature. Three unique solutions, labeled as 2a, 2b, and 2c, were obtained by dissolving 2 g of gelatin in 100 mL of three different solvents: water (as a control), 1% CS extract (containing the aqueous silverskin extract (sse) prepared at a concentration of 1% *w*/*v*), and 2% CS extract (containing the aqueous silverskin extract (sse) prepared at a concentration of 2% *w*/*v*), respectively. In order to produce hybrid films, three separate beakers were utilized, each containing 90 mL of S1. Subsequently, 10 mL of the three distinct solutions, namely 2a, 2b, and 2c, was added individually to each beaker. In the research conducted by S. Ahmed et al., in contrast to other compositions, it was discovered that the blended film with a concentration of CS:GL (90:10) displays excellent morphology [37]. The mixtures were subsequently placed on a magnetic stirrer without the use of heat. After 30 min, polyethylene glycol (PEG), acting as a plasticizer, was added to each beaker, at a concentration of 25% *w*/*w* relative to the total polymer mass (chitosan and gelatin). The contents of the beakers were then stirred for an additional 2 h. The final solution degassed using ultrasonication for 10 min and then placed onto flat glass plates. After that, the solvent was dried for 24 h at 40 °C in a vacuum oven, operating at a residual pressure of 50 mbar. The film fabrication process was performed in triplicate for all formulations.

### 2.4. Material Characterization

#### 2.4.1. Chemical Structure and Morphological Characteristics

The Perkin Elmer Spectrum 1 spectrophotometer (ATR) instrument was utilized for the FTIR measurements. Thin films were used for the measurements, and spectra were acquired at a resolution of 2 cm^−1^ spanning the range of 4000 to 650 cm^−1^ (32 scans).

The JSM 7610 FPlus (JEOL, Tokyo, Japan) FESEM scanning electron microscope was utilized. The instrument’s resolution was 5 nm. Samples must be conductive in order to be analyzed. To achieve this, a thin layer of carbon was applied to the samples in order to increase film surface conductivity.

#### 2.4.2. Physicochemical Properties

With a precision of 0.01 mm, the film thickness was measured with a handheld micrometer. Six replicates were carried out at random locations for every sample treatment.

A 1 cm × 1 cm piece of the film was cut and weighed both before and after it was heated for 24 h at 105 °C to find the Water Content (WC) of the films [38]. For every film, three duplicate measurements were taken. The following equation was used to estimate the percentage water content:

(1) $$Water\;content\left(\%\right)=\frac{M_{o}-M}{M_{o}}\times 100$$

where M is the bone-dry mass (g) and M_o_ the initial mass (g).

With some adjustments, the techniques outlined by Silva et al. [39] and Zhong et al. [40] were used to assess the degree of swelling and film solubility [41]. Film specimens measuring 1 cm × 1 cm were subjected to a drying process at a temperature of 70 °C for a duration of 24 h within a vacuum oven. This procedure aimed to obtain the initial dry mass (M_1_) of the films. Subsequently, the dried films were placed in 30 mL of distilled water. The beakers were wrapped in plastic wrap and kept at 25 °C for a full day in order to guarantee adequate containment. Following this period, any leftover water in the beakers was disposed of, and filter paper was used to superficially dry the remaining film specimens. These leftover film samples (M_2_) were subsequently dried in a vacuum oven for a further 24 h at 70 °C. The goal of this subsequent drying procedure was to ascertain the films’ ultimate dry mass (M_3_). For every film specimen, three measurements were made to guarantee precision and uniformity. The degree of swelling and film solubility were then computed as follows:

(2) $$Film\;Solubility=\frac{M_{1}-M_{3}}{M_{1}}$$

(3) $$Swelling\;degree=\frac{M_{2}-M_{1}}{M_{1}}$$

#### 2.4.3. Color Measurements

Measurements of the colorimetric indicators were performed using a Macbeth CE 3000 spectrophotometer under *D*_65_ illumination, 10° standard observer with ultraviolet (UV) and a specular component included. L*, a*, b*, c* (chroma), and h (hue) were measured. In addition, the color strength of the films was examined with the Kubelka–Munk equation (light reflectance technique):

(4) $$\frac{K}{S}=\frac{{\left(1-R\right)}^{2}}{2R}$$

where S and K are scattering and absorption coefficients, and R is the reflectance at the wavelength of maximal absorption.

#### 2.4.4. Thermal Properties by Differential Scanning Calorimetry (DSC)

Each synthesized material’s glass transition temperature and melting point temperature were ascertained using the Perkin-Elmer DSC-Diamond equipment (Waltham, MA, USA). After carefully weighing each sample, 5–6 mg were added to the standard Perkin-Elmer sample pan. After that, the pan was properly positioned inside the instrument and sealed. At a rate of 10 °C per minute, the samples were first heated to 200 °C. They were then chilled to −20 °C for two minutes at the same pace. The samples were then reheated to 150 °C at a rate of 10 °C min^−1^.

#### 2.4.5. Oxygen and Water Vapor Permeability

Using a gas permeability analyzer, the model N500 instrument (Guangzhou Biaoji Packaging Equipment Co., Ltd., Guangzhou, China), the oxygen permeability of the produced films was assessed under the following settings: constant temperature of 23 °C, 0% relative humidity, and gas flow rate of 10 mL/min.

Using glass Petri dishes that were 6 cm in diameter and 3 cm in height, filled with 10 mL of distilled water to generate an environment with 100% humidity, water vapor permeability was assessed in accordance with ASTM E96 [42]. Prior to being placed in a desiccator with active silica gel, the dishes’ original weights were noted. Weights were taken at regular intervals while the samples were kept in a desiccator for a whole day. For additional processing, the thickness of the film was measured at six random locations. The cup’s weight dropping suggested that water seeped through the film. The first step was to graph the water transmission curve over time and determine the slope (Δ*m*/Δ*t*) in its linear section in order to evaluate water vapor permeability.

The water vapor transmission rate (WVTR) and water vapor permeability were calculated with the following equations:

WVTR = (Δm/Δt)/A(5)

(6) $$WVP=\frac{WVTR\times d_{film}}{\Delta p}=\frac{\left[\left(\Delta m/\Delta t\right)/A\right]\times d_{film}}{\Delta p}$$

where A is the area of the film, d_film_ the film thickness, and Δp the differential pressure of water vapor between the two sides of the film (Τ = 20 °C, Δp = 2339 Pa).

#### 2.4.6. Antioxidant Activity

Utilizing the DPPH (2,2-diphenyl-1-picrylhydrazyl) method, the antioxidant activity was evaluated. A total of 6 mg of each film sample were put in a glass vial with three milliliters of DPPH solution and incubated for 48 h at 25 °C without any light. A UV spectrophotometer (Shimadzu Spectrophotometer UV-1800, Shimadzu, Kyoto, Japan) was used to measure the absorbance of the DPPH• reactant over a 400–700 nm range, with maximum absorbance recorded at 516 nm. With the absorbance of the DPPH solution acting as the control, the percentage of antioxidant activity was calculated to ascertain the films’ antioxidant activity (AA) using the given equation:

(7) $$AA\left(\%\right)=\frac{{ABS}_{control}-{ABS}_{sample}}{{ABS}_{control}}\times 100$$

#### 2.4.7. Mechanical Properties

According to ASTM D882 [43], tensile tests were conducted using an Instron 3344 dynamometer (Instron, Norwood, MA, USA) at a crosshead speed of 10 mm/min. A Wallace cutting press was used to cut dumb-bell-shaped tensile test specimens, with the central parts measuring 5 × 0.5 mm in thickness and 22 mm in gauge length. The mean values of Young’s modulus, tensile strength at yield, and tensile strain at break were calculated by averaging the results of at least five tests made for each sample.

### 2.5. Statistical Analysis

Every piece of data was examined three times. The properties of all the films under study were compared statistically using one-way analysis of variance (ANOVA). IBM SPSS Statistics 28 was used for statistical analysis. A *p*-value ≤ 0.05 was established as the threshold for statistical significance.

## 3. Results and Discussion

### 3.1. Morphological Characteristics

**Figure 2:**
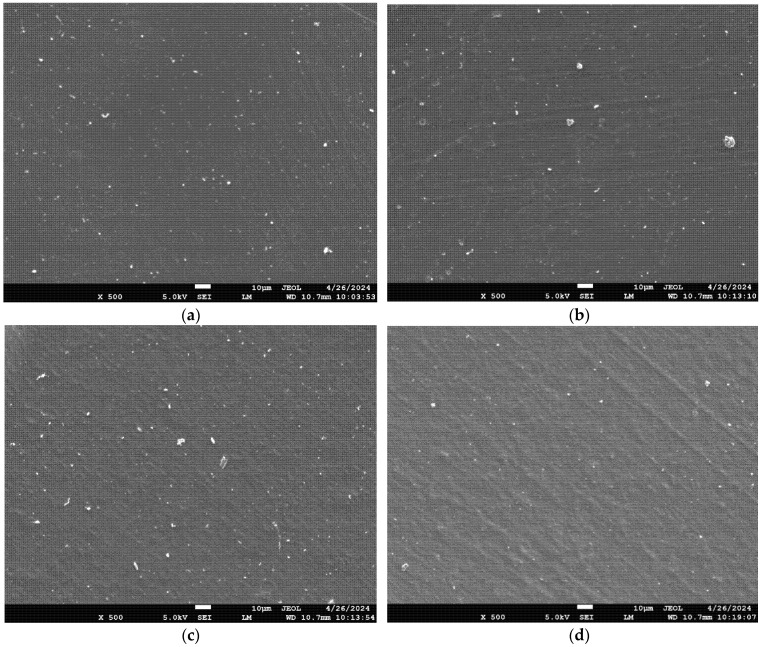
Scanning electron microscopy images of (**a**) pure chitosan, (**b**) chitosan–gelatin control (**c**) chitosan–gelatin-1%sse and (**d**) chitosan–gelatin-2%sse biocomposite films.

To study the surface morphology of the fabricated films, scanning electron microscopy (SEM) (Figure 2) was used. The morphology of each film is consistent, smooth, and homogeneous; there are no cracks or disruptions. The integration of CS extract was successfully accomplished, as the structure of films was not affected. A smooth, compact, and homogenous surface was also observed for pure chitosan–gelatin films in analogous research, although the incorporation of Ag nanoparticles modified the surface to a coarse and heterogeneous one, with agglomeration of particles [16]. In addition to being clear and homogeneous, blended CSGL films crosslinked with boric acid (BA) to enhance a number of characteristics also showed good component compatibility [37]. Additionally, the incorporation of polyethylene glycol as a plasticizer contributed to enhancing the flexibility of the films. It has been demonstrated that chitosan–gelatin mix films are homogeneous because of the high miscibility between the two biopolymers [5,44], which improves the blended films’ material qualities when compared to those made from the pure polymers.

### 3.2. Chemical Structure

**Figure 3:**
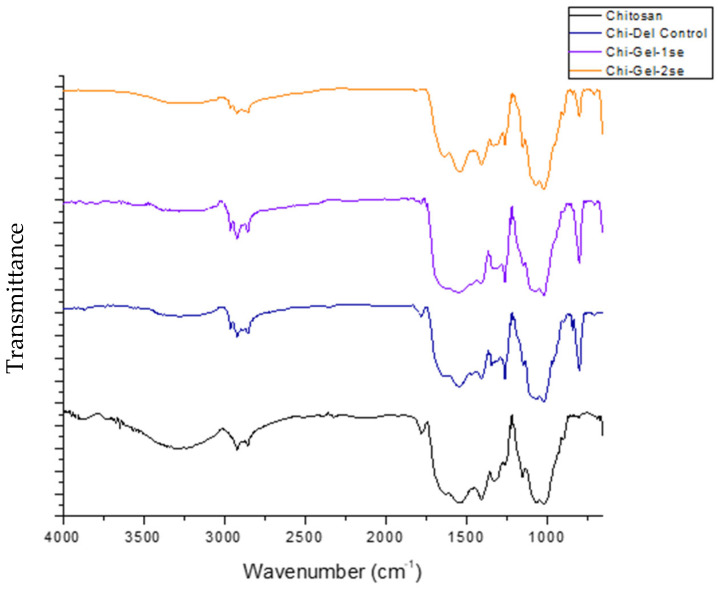
FTIR spectra of pure chitosan, chitosan–gelatin control, chitosan–gelatin-1%sse, and chitosan–gelatin-1%sse biocomposite films.

The FTIR spectra of pure chitosan, Chi-Gel-Control, Chi-Gel-1%sse, and Chi-Gel-2%sse films were carried out and are shown in Figure 3. The main characteristic peaks of chitosan at 3320 (broadband, -OH stretch), 2880 (C-H stretch), 1650 (C=O stretch), 1580 (N-H bend), 1378 (O-H bending), and 1030 (C-O-C stretch) cm^−1^. The findings from the CS film align well with previously documented results in the literature [45,46]. The intricate and distinct arrangement of hydrogen bonds, encompassing the OH, C-O, and NH groups, contributes to the overall complexity of the infrared (IR) spectra of chitosan. This complexity is further compounded by the broad nature of the IR bands commonly observed in natural polymers [47,48,49].

The spectra of the CS-GL film also showed a broad band at 3320 cm^−1^, which is linked to the stretching vibrations of pendant groups like NH_2_ and OH that are present in gelatin and chitosan. Furthermore, the asymmetric stretching vibrations of CH_2_ groups inside the chitosan chain are represented by the band at 2880 cm^−1^. Additionally, peaks at 1250 cm^−1^ (C-N stretching), 1580 cm^−1^ (N-H bending), and 1650 cm^−1^ (C=O stretching) were noted. Lastly, the polysaccharide chain’s C-O-C stretching is connected to the absorption peak at 1030 cm^−1^. Furthermore, a new band at 550 cm^−1^ is discernible, indicating the development of coordination links between different gelatin and chitosan groups. The findings for the Chi-Gel film align well with the results documented in previous studies [16,46,50].

### 3.3. Physico-Chemical Characterization

**Figure 4:**
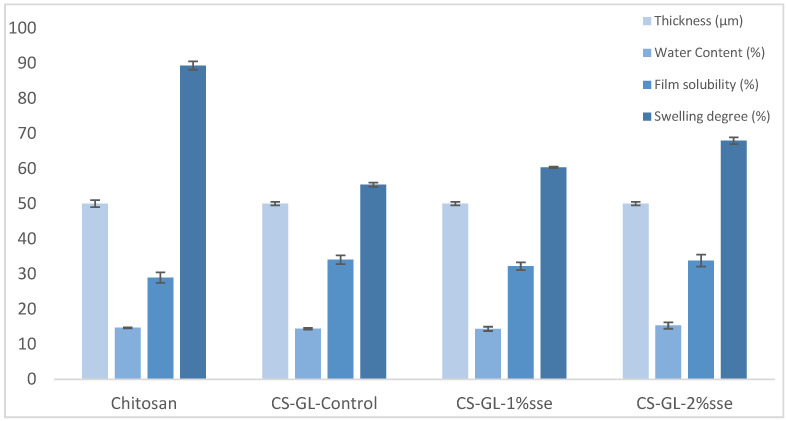
Physico-chemical characterization of hybrid biocomposites.

Table 1 and Figure 4 displayed the fundamental film characteristics, such as thickness, water content, film solubility, and degree of swelling. All films had the same thickness. Similar values of water content were observed for all films, except for Chi-Gel-2%, which was 6.1% higher in comparison to the control film. According to a similar study, GL blends cause chitosan films’ moisture content decreasing [37]. WC values of Chi-Gel films in this study were lower than those in this study, up to 30.7%. As PEG increases the intermolecular gaps between polymeric chains, more room is available for collecting moisture in plasticized films, which can result in higher water content values [50]. Higher values of WC observed in chitosan/starch films [51].

The ability of biodegradable films to resist water can be greatly affected by two important factors: solubility and swelling. These features are essential in evaluating the film’s ability to resist water infiltration, particularly in high-humidity conditions [41]. The solubility value, which is correlated with the hydrophilicity of films, is a measurement of the films’ resistance to water [52]. Table 1 demonstrates that the chitosan film displayed minimal solubility, which increased up to 17.74% when gelatin was added to the matrix. At first, adding CS extract to the matrix reduced the films’ water solubility by up to 5.14%; at 2%, the results were comparable to those of the control film. The increase in solubility is due to the gelatin content to the matrix, which is a natural product and retains moisture, after being immersed in water, thus allowing more water to be absorbed. The coffee silverskin extract did not affect the solubility values. The same results were also found in similar investigations. Given that gelatin is hydrophilic or because it is soluble, blended films were marginally more soluble than pure chitosan films [37]. The solubility values were lower in that study than ours. Gelatin/chitosan nanocomposite reinforced by NiO nanoparticle films also exhibited lower values of solubility. The solubility of gelatin/chitosan nanocomposite films with Ag nanoparticles was observed to be higher, with values ranging from 42.92 to 52.60% [14].

The quantity and kind of intermolecular chain interactions have a significant impact on a polymer material’s degree of swelling [53]. Swelling degree showed a reduction of 37.99% for the control film, in comparison to pure chitosan film. An increase of up to 8.9% was observed, when 1% coffee silverskin extract added to the matrix, and up to 22.6% for Chi-Gel-2% sse, with respect to the control film. Chitosan–gelatin edible films made by combining *Dracocephalum kotschyi* (D. kotschyi) essential oil nanoemulsion were found to have higher swelling degree and water content values as well as comparable solubility [18]. Swelling degree values were comparable for films containing dialdehyde carboxymethyl cellulose and different amounts of coffee leaf extract in the gelatin matrix [12].

### 3.4. Color Measurements

**Figure 5:**
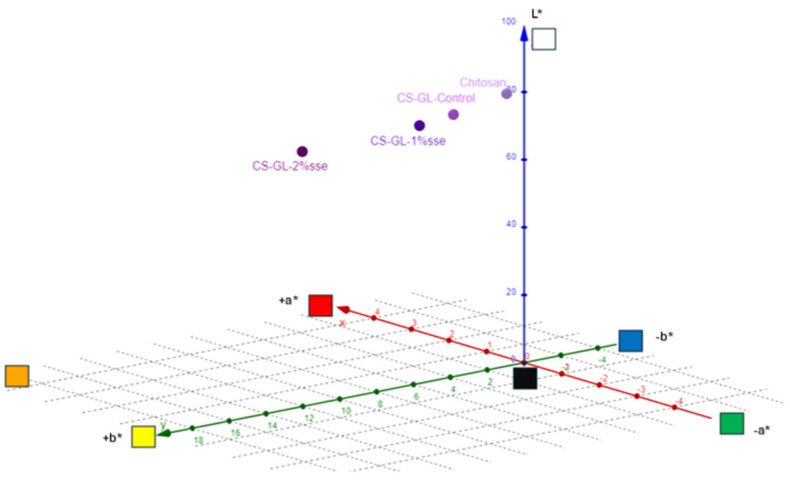
Observation of change in color measurements.

Since color greatly affects the container’s aesthetics, it is an important consideration when choosing polymeric materials for packaging production. Additionally, color choice can also impact the see-through property and light transmission capabilities of the packaging [54]. The alteration in film color following the introduction of CS extracts, as illustrated in Figure 5 and Table 1, prompted an assessment of the color properties of the films. Compared to the pure chitosan film, the Chi-Gel composites showed a decrease in lightness (L*) values. All samples showed a steady reduction in lightness values, which decreased by 3.07% when gelatin was added to the matrix and by 5.71% when CS extract was added at the highest concentration. Adding CS extract or gelatin to the matrix had no effect on the a* results. However, the b* and c* values for the films initially increased by 63.62% when gelatin was added to the matrix and doubled when coffee silverskin water extracts were added to the matrix. The values of H* decreased with the addition of gelatin to the chitosan film matrix in comparison to the pure chitosan film, showing a reduction of up to 10.23%. Conversely, the values remained constant when coffee silverskin extract was added. As the concentration of coffee silverskin extract increased in the matrix, a yellowish appearance of the film was observed. The progressive inclusion of extracts resulted in enhanced color strength values, with particularly elevated levels for the Chi-Gel-Control film. The Chi-Gel-Control film presents similar values of lightness and lower values of a* and b* in comparison to an analogous investigation [16]. PLA films acquired a brown hue after the incorporation of coffee and cocoa extracts, exhibiting different levels of intensity based on the extract type and concentration. Among the films, those infused with coffee extract displayed the lightest shade, whereas films infused with cocoa extract showcased the darkest tone [55]. The K/S values show variations in the color intensity of the films. Higher values indicate stronger absorption and higher concentration of pigment due to the CS extract, while lower values are indicative of “faint” hues or higher scattering.

### 3.5. Thermal/Calorimetric Properties

DSC curves were recorded during two heating cycles and one cooling cycle. Pure chitosan films showed Tg at 97.5 °C and Tm at 126.04 °C in the initial scan (Table 1). The outcomes can be ascribed to an unstructured state that remained unaffected by the existence of water molecules [56]. No evidence of cold crystallization was observed during the cooling process, possibly due to very low sample crystallinity, and the second scan did not reveal any glass transition rate or melting point for pure chitosan film. Two thermal events have been documented in pure chitosan films during the first heating cycle, specifically a glass transition temperature (Tg) at 54 °C and a melting temperature (Tm) at 88 °C [56]. These events are attributed to the presence of partially crystalline acetamide-rich regions within the films, which facilitate the mobility necessary for the release of trapped acetic acid. Chitosan films have shown varying Tg values within the temperature range of 60–93 °C, with the specific value depending on the type of acid utilized during film preparation, such as butyrate, propionate, acetate, and formate [57]. The absence of evidence for a transition temperature has been observed by researchers, suggesting that the chitosan Tg may be located at a temperature above the one used in their studies (220 °C) [58,59]. Lower values of Tg at 51.7 and Tm at 70.6 °C in the first scan were observed for pure chitosan by Bonilla et al. [17].

In the first scan, the chitosan–gelatin control film showed Tg at 94.5 °C and Tm was not detected. Similar findings were observed for films based on chitosan–gelatin and coffee silverskin extract, with no melting point and glass transition temperature at the same values as the control film in the first scan. Throughout the process of cooling, an exotherm phenomenon was noted for all chitosan–gelatin hybrid films. Cold crystallization at −23.58 °C was witnessed in the control film, as well as slightly elevated values for films with CS extract 1% and 2% at −17.95 °C and −15.13 °C, respectively. When CS extracts were incorporated, the crystallinity values significantly decreased. In the second scan, only the melting point was observed at 50.24–52.03 °C for all hybrid films. The blended films’ thermal stability was impacted since the values they displayed were lower than those found in the pure chitosan film. This might be explained by the possible interference with the chitosan matrix’s ability to develop a semi-crystalline structure. No significant differences were observed when CS extract was added to the matrix. The increase in chitosan proportion from 50:50 chitosan/gelatin composites to 75:25 chitosan/gelatin exhibited lower values of Tg and Tm in the first scan and further down values of Tg in the second scan, due to a fraction abundant in glycerol and another fraction rich in chitosan [17]. Furthermore, the glass transition temperature was also affected when plant ethanolic extracts were mixed with the matrix. A decrease in thermal stability resulted from the greater inclusion of green and black tea extracts, which did not cause any changes in the endothermic peaks that tended to shift towards lower temperatures as the concentration of tea extracts increased [41].

### 3.6. Antioxidant Activity

**Figure 6:**
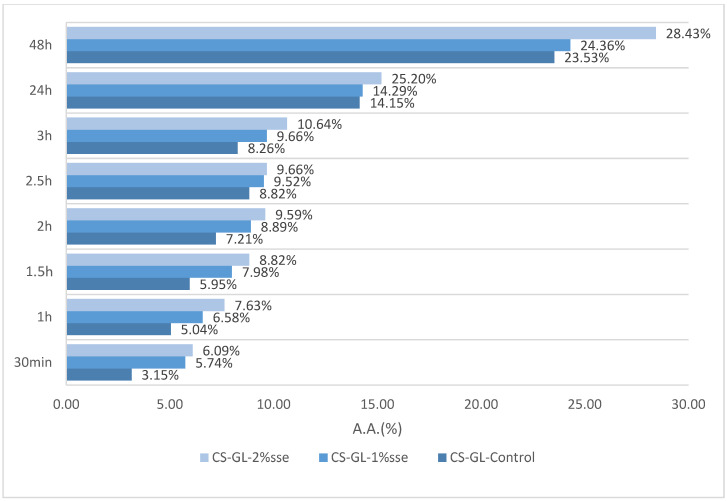
Observation of antioxidant activity of all hybrid biocomposite films.

By the DPPH method, a frequently used approach in evaluating the antioxidant efficacy of packaging films, the stability of the produced materials was assessed to ascertain their antioxidant characteristics [60]. Antioxidants are very beneficial in the field of food packaging materials as they may effectively combat the negative effects of free radicals. Thus, they contribute significantly to protecting against spoiling and the degradation of vital nutrients [61]. The initial color of the samples was purple, which subsequently transformed into yellow, thereby indicating the existence of antioxidants. The UV absorbance was measured to determine the extent of the color change, and the results are shown in Figure 6.

As anticipated, all materials demonstrate a peak at 516 nm. The inclusion of coffee silverskin extracts in the films resulted in a progressive enhancement of their antioxidant activity, corresponding to the concentration of the extracts. Furthermore, the Chi-Gel-Control film exhibited antioxidant activity. Notably, the films containing coffee silverskin extract displayed a greater antioxidant capacity, with the highest observed in the Chi-Gel-2%sse film, reaching up to 28.43% and being 20.82% higher than Chi-Gel-Control after 48 h. The predominant origin of antioxidative potential in coffee silverskin is linked to the existence of polyphenols. These polyphenols consist of a wide array of substances that exhibit the capability to neutralize free radicals. Coffee silverskin, additionally, holds a variety of bioactive components such as melanoidins and caffeine, which have the potential to enhance the antioxidative properties [62]. In a detailed study [63], a high amount of caffeine was observed in CS extracts in different solvents, together with polyphenols, such as phenolic acids (caffeoylquinic acids), vanillic acid, and small amounts of flavonoids, such as quercetin. It should be noted here that to clearly correlate the observed antioxidant activity with the compounds found in CS extracts, a detailed analysis is required using analytical techniques such as HPLC/LC-MS. However, this would require a large amount of extra experimental work and the main goal of this research would be lost. Therefore, we assume that the compounds responsible for the observed antioxidant activity are those measured by these authors [64].

Therefore, it has been confirmed that the use of CS extract as a filler in a polymeric matrix gives the film antioxidant properties. Gelatin-based films containing dialdehyde carboxymethyl cellulose and coffee leaf extract also showed superior antioxidant properties, which were linked to the extract’s potent phenolic components [12]. When Yong et al. [48] investigated the antioxidant characteristics of chitosan/anthocyanin-rich eggplant extract, they likewise found an enhancement in antioxidant activity [65]. Additionally, after S-chitin enrichment, the antioxidant efficiency of CS/Gel-based films was markedly increased. Pure coffee silverskin as an additive in a polymer matrix led to remarkable antioxidant characteristics of films, due to the higher DPPH radical scavenging activity and oxidation induction times (OIT) [65,66]. The antioxidant efficacy of chitosan/gelatin films also increased with a rise in Ag nanoparticle concentration [14]. These results show the potential for use as an antioxidant food-packaging material using chitosan-based films containing gelatin and CS extract.

### 3.7. Gas and Water Vapor Permeability

The ability of a material to limit the permeability of gas and water vapor is referred to as its material barrier characteristics. The shelf life of food packaging is influenced by two essential material specification qualities, namely the water vapor transmission rate and the oxygen transmission rate. The results of the tests performed on the samples and the measurement of the water vapor permeability and the water vapor and oxygen transmission rates are shown in Table 1.

The WVTR and WVP measurements were unaffected by the addition of coffee silverskin extracts. This lack of significant change may result from a balance between hydrophilic compounds, which facilitate water diffusion, and hydrophobic polyphenols or lipophilic constituents, which hinder it by locally densifying the polymer matrix, effectively counterbalancing each other. A characteristic known as water vapor permeability suggests that water molecules are soluble in water and diffuse through the film’s matrix. A number of variables, including the molecular weight of the polymers, the kind and amount of crosslinking, and the preparation circumstances that affect the network structure inside polymeric films, can affect the water vapor permeability of films [52]. It has been demonstrated that the interaction between two components causes the WVP of CSGL films to decrease when compared to that of pure CS films [37]. Studies have demonstrated that the presence of plasticizer leads to a reduction in intermolecular interaction, resulting in an expansion of molecular spaces between polymer matrixes. In the end, this expansion increases the plasticized films’ permeability [54,56]. Intermolecular interactions decrease when the plasticizer concentration rises because the molecular gaps among polymer chains also expand. This decrease in interactions subsequently diminishes the mechanical strength of the films while simultaneously increasing their flexibility [67,68]. Edible films made by combining a nanoemulsion of Dracocephalum kotschyi essential oil to the Chi-Gel matrix showed higher WVP values [18]. Lower values of WVP were observed for CS/Gel-based films integrated with sulfur-functionalized chitin.

The integrity of the film, the ratio of crystalline to amorphous areas, the hydrophilicity/hydrophobicity, and the mobility of polymeric chains are some of the variables that affect the gas permeability of edible films and coatings. Furthermore, a plasticizer or other additives and the interaction of the film-forming polymer are important factors in determining the permeability of the film [5]. Oxygen plays a crucial role in triggering oxidation, leading to various undesirable alterations in food properties like smell, appearance, taste, and nutritional content. As a result, using films with strong oxygen barriers can improve food quality and increase shelf life [69]. We observed that there is a decrease in the oxygen transmission rate as the percentage composition of coffee silverskin extract increases. The observed reduction in OTR upon incorporation of coffee silverskin extract may be attributed to multiple factors. Polyphenolic compounds and other bioactive constituents in the extract can interact with the polymer chains, increasing intermolecular interactions and leading to structural densification of the film matrix. This denser network reduces the free volume and limits oxygen diffusion through the film. Additionally, hydrophobic components in the extract, such as fatty acids or lipophilic compounds, can further impede gas permeability by creating tortuous pathways for oxygen molecules, thereby enhancing the film’s barrier properties. Similar mechanisms have been reported in biopolymer films containing plant extracts, which combine structural reinforcement with selective molecular interactions to decrease gas transmission. The OTR value decreased by 28.18%, when coffee silverskin extract added at 1% and 53.64% when coffee silverskin extract was 2% in the matrix, compared with the control film. Additionally, it has been noted that adding fatty acids, like stearic acid, can successfully lower the rate of gas transmission [70]. Pure gelatin films showed higher values of OTR and gelatin films based on dialdehyde carboxymethyl cellulose and varying contents of coffee leaf extract presented similar values in comparison to our findings [12]. PLA films incorporating coffee extract exhibited a more significant decrease in oxygen permeability when compared to PLA films incorporating cocoa extract [55].

### 3.8. Mechanical Properties

**Figure 7:**
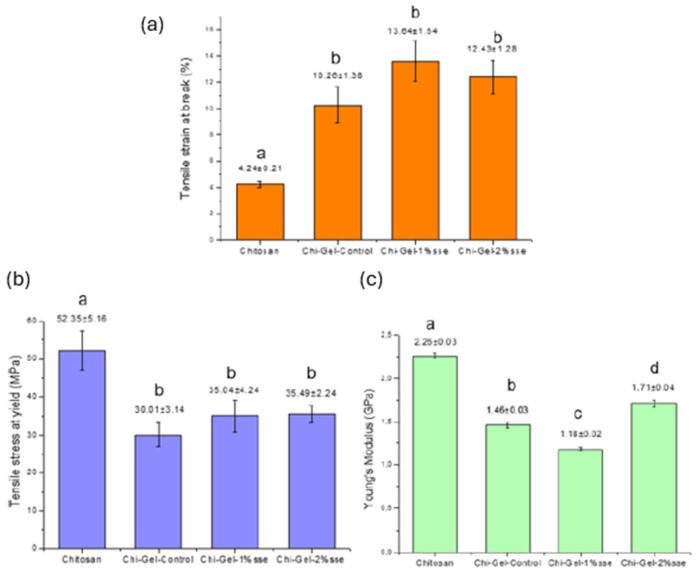
Mechanical properties of all biocomposites: (**a**) tensile at strain at break (%), (**b**) tensile stress at yield (MPa), and (**c**) E-Modulus (GPa) (different lower case letters in each figure denote statistically different values).

Measuring a film’s extensibility and tensile strength is essential for figuring out how well it can tolerate environmental stressors that are frequently present in packaging applications. Given their direct influence on product performance and market acceptance, studying the mechanical characteristics of films is extremely important. Figure 7 presents the outcomes of mechanical tests. The blending of Chi with Gel increased tensile strain up to 142% in comparison to pure chitosan film. The addition of CS extract to the matrix led to higher tensile strain, up to 33% with respect to pure chitosan–gelatin blending and 221.7% compared to neat chitosan film. This pronounced improvement may indicate a plasticizing effect induced by the extract. Bioactive constituents such as polyphenols can disrupt intermolecular interactions within the polymer network, thereby decreasing chain–chain attraction and increasing molecular mobility, which results in enhanced film flexibility and greater elongation at break. The elongation at break of Chi-Gel-Control was lower compared to similar films (17.99 ± 0.68%) [16]. The incorporation of chitosan with gelatin enhances the mechanical properties of films because of electrostatic attractions among the diverse functional groups present in each component [70]. All Chi-Gel blended films showed lower values than pure chitosan film, while Chi-Gel-1%sse and Chi-Gel-2%sse films demonstrated higher values of tensile stress at yield than the control, by up to 18.26%. Lower values of tensile stress for Chi-Gel-Control were observed by Ahmed et al. (20 MPa) [37] and similarly by Kumar et al. (28.87 ± 0.49 MPa) [16]. As shown in Figure 6, the E-Modulus of GS-GL films was up to 35.4% lower than that of chitosan, with comparable values. The E-Modulus of Chi-Gel-2% sse exhibited a significant increase of up to 18.26% when compared to Chi-Gel-Control.

Compared to the chitosan/gelatin films containing sulfur-functionalized chitin made by A. Khan et al., the CS-GL and coffee-silverskin extract films made in the present study showed a higher tensile strain at break, lower tensile stress, and comparable E-Modulus values (E-Modulus 1.9–2.2 GPa, tensile strain 4.5–6.4%, and tensile stress 46.7–55.3 MPa). Sun and colleagues found that gelatin-based multifunctional composite films combined with dialdehyde carboxymethyl cellulose and coffee leaf extract films had lower values of tensile strain (%) and higher values of tensile strain and E-Modulus [12]. Gelatin/chitosan nanocomposites films reinforced by NiO nanoparticles exhibited lower values of tensile stress (ranging from 12.32 to 26.61 MPa) and higher tensile strain (ranging from 16.84% to 20%). The tensile stress and tensile strain of gelatin/chitosan nanocomposite films with Ag nanoparticles were observed to be lower, with values ranging from 24.39 to 26.40 MPa and 4.12% to 4.50%, respectively [14]. Our composite films’ mechanical properties are superior to those of LDPE, which normally has strengths between 22 and 23 MPa, and comparable to those of plastic films like HDPE, which normally have strengths between 19 and 44 MPa [71]. A more robust comparison with conventional plastics further contextualizes the performance of the developed films. Recent work by Silva et al. [72] demonstrated that modified chitosan-based biopolymer films could achieve tensile strengths and ductility values approaching those of LDPE and HDPE, highlighting the potential of polysaccharide systems to compete with petroleum-derived materials. In line with these findings, the CS-enriched Chi–Gel films produced in the present study exhibit tensile strain and strength values within the lower-to-intermediate range of commercial polyethylene grades, supporting their suitability as sustainable packaging candidates. It is worth noting that dissolving chitosan in acetic acid can weaken its inherent hydrogen-bonding network and partially disrupt interchain interactions, as reported in previous studies [73]. Under these conditions, the improvements observed in tensile strength, elongation at break, and OTR may not derive solely from the effects of gelatin and coffee silverskin extract. Rather, these components may also contribute to restoring or reinforcing the structural integrity of the chitosan matrix that becomes compromised during acid dissolution. This perspective provides an additional explanation for the enhanced mechanical and barrier properties of the developed films.

## 4. Conclusions

In this research, we fabricated hybrid biocomposites of chitosan–gelatin with coffee silverskin extract, including glycerol as a plasticizer, using the solution casting method. The films exhibited uniformity in their structure and as the concentration of coffee silverskin extract increased in the matrix, a yellowish appearance of the film was observed. The results from the solubility experiments demonstrated that the composite films exhibited higher solubility compared to the pure chitosan film. Furthermore, it was observed that the water content was not affected by the addition of gelatin or coffee silverskin extract added to the matrix and swelling degree of the composite films increased as the quantity of extract within the composite matrix increased, but was still lower than pure chitosan. The enhanced thermal stability of the biocomposite films was confirmed by the DSC technique. The incorporation of CS extract significantly enhanced the antioxidant activity of the films. The enhanced mechanical performance was primarily due to increases in tensile strain at break and tensile stress at yield, likely resulting from electrostatic interactions among the polymer chains. Adding the extract to the matrix caused a reduction in the oxygen transmission rate, with no impact on the water vapor permeability. Therefore, this study suggests that the developed chitosan–gelatin hybrid biocomposite films with coffee silverskin extract have potential as a new biomaterial for food packaging systems.

## Figures and Tables

**Table 1:** Properties of hybrid biocomposites.

	Chitosan	Chi-Gel-Control	Chi-Gel-1%sse	Chi-Gel-2%sse
Physico-chemical characterization				
Τhickness (μm)	50 ± 1.0	50 ± 0.5	50 ± 0.5	50 ± 0.5
Water content (%)	14.67 ± 0.14 ^a^	14.43 ± 0.24 ^a^	14.36 ± 0.61 ^a^	15.31 ± 0.90 ^a^
Film solubility (%)	28.92 ± 1.49 ^A^	34.05 ± 1.24 ^B^	32.3 ± 1.09 ^B^	33.79 ± 1.73 ^B^
Swelling degree (%)	89.33 ± 1.19 ^α^	55.39 ± 0.59 ^β^	60.32 ± 0.21 ^γ^	67.91 ± 0.98 ^δ^
Color measurements				
L*	90.65	87.87	86.46	85.47
a*	−1.84	−1.89	−1.87	−1.84
b*	4.7	7.69	9.49	15.79
c*	5.05	7.91	9.65	16.03
h	111.4	100	100.4	103.6
R% (400 nm)	63.05	44.99	48.82	48.16
K/S	0.11	0.34	0.27	0.28
Thermal properties				
Tg (°C) (1st heat)	97.53	94.62	95.18	93.82
Tm (°C) (1st heat)	126.04	-	-	-
DH (J/g) (1st heat)	677.85	-	-	-
Tcc (°C) (cooling)	-	−23.58	−17.95	−15.13
DH (J/g) (cooling)	-	−15.27	−15.87	−13.85
Tm (°C) (2nd heat)	-	50.24	51.47	52.03
DH (J/g) (2nd heat)	-	16.33	21.54	20.60
Permeability properties				
WVTR (g/m^2^∙d)	14.01	13.69	13.52	13.69
WVP (10^−7^) (g/m∙d∙Pa)	2.99	2.93	2.89	2.93
OTR(cm^3^/(m^2^∙d∙0.1 MPa)	7.50	6.60	4.74	3.06

Different superscript letters denote statistically different values.

## Data Availability

The original contributions presented in this study are included in the article. Further inquiries can be directed to the corresponding author.
